# Enhancing perovskite solar cells efficiency via dual surface passivation

**DOI:** 10.1371/journal.pone.0351439

**Published:** 2026-07-10

**Authors:** Refka Sai, Shrouq H. Aleithan

**Affiliations:** 1 Department of Physics and Astronomy, University of Carthage, Carthage, Tunisia; 2 Department of Physics, College of Science, King Faisal University, Al-Ahsa, Saudi Arabia; Universidad Autónoma de Querétaro: Universidad Autonoma de Queretaro, MEXICO

## Abstract

Effective defect passivation is essential for achieving high performance in perovskite solar cells (PSCs). Dimensional engineering provides a powerful strategy to suppress non-radiative recombination in both the bulk and surface regions of PSCs. In this work, we present a novel interfacial passivation approach for the perovskite/hole transport layer interface using a dual-cation passivation layer composed of guanidinium bromide (GuaBr) and n-phenylethylammonium bromide (n-PEABr). This dual-cation strategy delivers an open-circuit voltage of 1.23 V and a power conversion efficiency (PCE) of 25.11%, significantly outperforming devices based on single-cation passivation. The combined cations induce the formation of a mixed 1D/2D perovskite structure, resulting in a more uniform and hydrophobic surface compared with unpassivated films. Moreover, stability tests conducted under ambient conditions (80% relative humidity) and continuous light-soaking reveal markedly enhanced device stability. The results demonstrate the superior passivation effectiveness of phenylethylammonium compared with previously reported methods. In particular, this approach surpasses the 23% PCE achieved using octylammonium passivation, achieving efficiencies exceeding 25%. Overall, the excellent defect passivation and favorable optical and electrical properties of phenylethylammonium play a key role in significantly improving both the efficiency and stability of PSCs.

## 1 Introduction

Perovskite solar cells (PSCs), which combine organic and inorganic materials, have garnered significant interest due to their cost-effective production, ease of solution-based manufacturing, and impressive power conversion efficiency (PCE) [1–4].Currently, the power conversion efficiency (PCE) of PSCs has surged from an initial3.8% to an impressive 25.7% [5–10]. This remarkable improvement is largely due to the distinct characteristics of perovskite materials (ABX_3_, where A, B, and X can be monovalent cations like CH^3^NH^3+^ MA^+^,FA^+^,Cs^+^, divalent metal cations likePb^2+^ andSn^2+^, and halogen anions such as ${\text{I}}^{-}$, ${\text{Br}}^{-}$, and ${\text{Cl}}^{-}$), which exhibit long carrier diffusion lengths and high optical absorption coefficients [11–15]. Nevertheless, there remains considerable room for enhancement compared to the theoretical Shockley–Queisser limit of approximately 33% [16,17]. A significant limitation arises from the inherent presence of various shallow-level defects (e.g., iodide and monovalent cation vacancies like VI, VMA, VFA, etc.) and deep-level defects (e.g., substitutions like MAI^+^, FAI^+^,IMA^+^,IFA^+^, etc., and uncoordinated Pb^2+^) within perovskite films fabricated through conventional solution processes [1–6,11,12,16–19]. These defects give rise to numerous carrier complex centers, ultimately compromising the performance of PSCs [20]. However, the underdeveloped methodologies and the exorbitant expenses associated with single-crystal PSCs do not align with the requisites of sustainable advancement. An alternative approach frequently adopted involves implementing post-passivation treatments aimed at mitigating defects.The rapid crystallization of perovskite throughout the processing in solution introduces structural imperfections and trapping sites within the device architecture, serving as sites for non-radiative electron-hole recombination. Recombination, a primary contributor to voltage drop in PSCs, can occur via three passages: within the perovskite bulk, at interfaces between perovskite and electron or hole transport layers (ETL or HTL), and through shunt pathways between ETL and HTL [21]. While frequent lattice imperfections within the perovskite bulk typically exhibit energy levels proximal to the conduction or valence bands, resulting in minimal recombination rates, highly efficient PSCs generally experience negligible recombination via shunt paths [22]. Consequently, recombination at interfaces at the perovskite/interfaces of the transport layer emerges as the predominant mechanism for carrier loss [23]. Consequently, extensive research efforts have focused on passivating the interfaces among the perovskite absorber and charge transport layers (CTLs) [24–30].Passivation methods can be categorized into physical and chemical approaches. Material passivation involves spatially segregating issues related to minority charge carriers within the device, while chemical passivation aims to reduce the number of imperfection/ trap states [31]. Chemical passivation encompasses various techniques including manufacturing techniques [32], additive integration [33], constituents manipulation [34], dimensional adjustment [35], and interface modification [36]. Dimensional engineering, particularly, garners considerable attention due to its significant enhancement of power conversion efficiency (PCE). This method involves altering the conventional 3D ABX3 perovskite atomic lattice by introducing bulky organic cations that facilitate the formation of low-dimensional columnar (1D) or layered (2D) crystal structures. Consequently, a mixed 1D–3D [34,37,38] or 2D–3D [39–43] perovskite structure emerges, which has the capacity to bolster device efficiency and stability. 2D perovskites are further categorized as Ruddlesden–Popper (RP) [44], Dion–Jacobson (DJ) [45], and alternating cations in the interlayer space (ACI) forms [46]. The ACI form exhibits reduced binding energy of excitons, extended duration of carrier persistence, and narrower or Similar optical bandgap to DJ and RP phases [47].To achieve Surface treatment using a one-dimensional or two-dimensional capping layer, a two-step process is typically employed. Firstly, a perovskite photoactive film is deposited, subsequently the deposition of a chloroform solution or an isopropyl alcohol (IPA) containing 1D/2D cations (referred to as spacer cations or interlayer) [48,49]. These spacer cations commonly consist of long-chain alkyl or aryl ammonium cations like cyclopropylammonium (CA^+^) [50], phenylethylammonium (PEA^+^) [50], methylammonium (MA^+^) [51], octylammonium ()A^+^) [52,53], and butylammonium (BA^+^) (BA+) [52,54,55]. Surface modification using bulky alkyl-ammonium spacer cations has shown promise in effectively mitigating defects, alongside enhancing stability in ambient conditions owing to their hydrophobic properties [54–57]. According to recent findings [58], a power conversion efficiency (PCE) of 23.38% was achieved for a perovskite solar cell passivated with octylammonium bromide (OABr).Yet, the stability performance remains lacking, as no stability data were presented for the passivated devices. Furthermore, in place of extended alkylammonium cations, scientists have explored using compounds containing guanidinium $({\text{CNH}}_{2}{)}^{3+}$ cations as the surface passivation layer between the transport layers and perovskite. In contrast to extended alkylammonium cations, guanidinium cations have been noted for their ability to partially infiltrate the perovskite bulk due to their smaller size, thereby providing bulk passivation. Chavan et al. [59] achieved a 20.07% efficiency employing GuaI for passivation at the perovskite/hole transport layer (HTL) interface. Similarly, GuaBr was employed as a surface passivation layer atop the perovskite in an alternate configuration, resulting in a certified open-circuit voltage $\left({\text{V}}_{OC}\right)$ of 1.175 V. Additionally, guanidinium thiocyanate was utilized using it for passivation at the perovskite/HTL interface in a flat structure, leading to a power conversion efficiency (PCE) about of 16.37% [60–65]. Most studies focusing on dimensional engineering of perovskite solar cells (PSCs) have typically utilized a single spacer cation either for bulk or surface modification. However, a number of investigations have explored dimensional engineering by employing combinations of multiple spacer cations [66–70]. These studies have highlighted that incorporating multiple alkylammonium cations can potentially enhance device performance by improving charge extraction, promoting growth and uniform nucleation of perovskite crystals, and reducing exciton binding energy [67–69]. Thus, there is significant value in exploring different utilizing various types of spacer cations to enhance surface passivation in PSCs. In our study, we demonstrate efficient surface modification of perovskite solar cells (PSCs) at the interface between the perovskite and hole transport layer (HTL) using a dual addition of two distinct spacer cations: phenylethylammonium bromide (PEABr) and guanidinium bromide (GuaBr). Extended alkylammonium cations have been recognized for enhancing device achievement by chemically passivating undercoordinated bonds on the perovskite surface, thereby creating a physical barrier that reduces ion extraction from the absorber material. Additionally, their hydrophobic properties contribute to improving the stability of the device in ambient conditions. In contrast, guanidinium salts have been observed to enhance cell achievement by promoting the creation of well-ordered crystal grains and compact within the perovskite layer, which minimizes recombination sites. Guanidinium ions can penetrate into the perovskite bulk and localize at grain boundaries (GBs), where they interact through hydrogen bonding with iodine species lacking full coordination, thereby suppressing charge recombination and ion migration.Our results indicate that PSCs treated with a combination of PEABr and GuaBr outperform devices treated with single spacer cations in terms of short-circuit current density $\left({\text{J}}_{SC}\right)$, power conversion efficiency (PCE), and stability. Within the dual-cation passivation layer, the PCE enlarged from 21.37% for the control cell to 25.11% for the passivated device, demonstrating significantly enhanced long-term stability as well. This research is motivated by the findings of Qi Jiang [71], Naeimeh Mozaffari [72], and Medhat M. Osman [73]. Jiang demonstrated that surface passivation with phenylethylammonium significantly improves perovskite solar cell efficiency by reducing defects and suppressing non-radiative recombination, achieving a Power Conversion Efficiency (PCE) of 23.32%. Osman also showed that passivation with n-octylammonium enhances PCE to 20.2%. Meanwhile, Mozaffari reported a PCE of 23.13% using a binary surface passivation approach with guanidinium and octylammonium spacer cations. Building on these insights, we aim to replace octylammonium with phenylethylammonium in the dual surface passivation of perovskite solar cells to further enhance their performance.

## 2 Materials and methods

### 2.1 Materials and layer preparation

To evaluate the impact of GuaBr, PEABr, and their combination on device achievement, we fabricated perovskite solar cells (PSCs) under three different conditions. These included as well as variations with passivation layers comprising GuaBr, PEABr, and a blend of both. To optimize the ratio between PEABr and GuaBr, we tested three different volume ratios: 1:1 (referred to as 1P-1G), 2:1 (labeled as 2P-1G), and 1:2 (designated as 1P-2G).The device architecture for these PSCs consisted of glass/FTO/compact TiO_2_ ${\text{cp-TiO}}_{2}$/mesoporous TiO_2_ (${\text{mp-TiO}}_{2}$)/polymethyl methacrylate (PMMA): phenyl-C60-butyric acid methyl ester (PCBM)/${\text{(MACl)}}_{0.33}{FA}_{0.99}{MA}_{0.01}Pb(I_{0.99}{Br}_{0.01}{)}_{3}$ perovskite/passivation layer/spiro-OMeTAD/Au.Subsequent to applying the perovskite precursor solution via spin-coating on the substrate and annealing at 150 °C for 25 minutes, the passivation solution was spin-coated onto the substrate, subsequent to an additional annealing step at 150 °C for 10 minutes. We monitored the devices over time and observed a slight color change toward a brown–yellow appearance after extended exposure. This change indicates the onset of degradation, which is commonly associated with the partial transformation of the perovskite phase under ambient conditions. Although systematic outdoor stability tests were not performed, these observations suggest that environmental factors such as moisture, oxygen, and light can affect the long-term stability of the fabricated PSCs. Further detailed stability studies will be conducted in future work.

**Fig 1:**
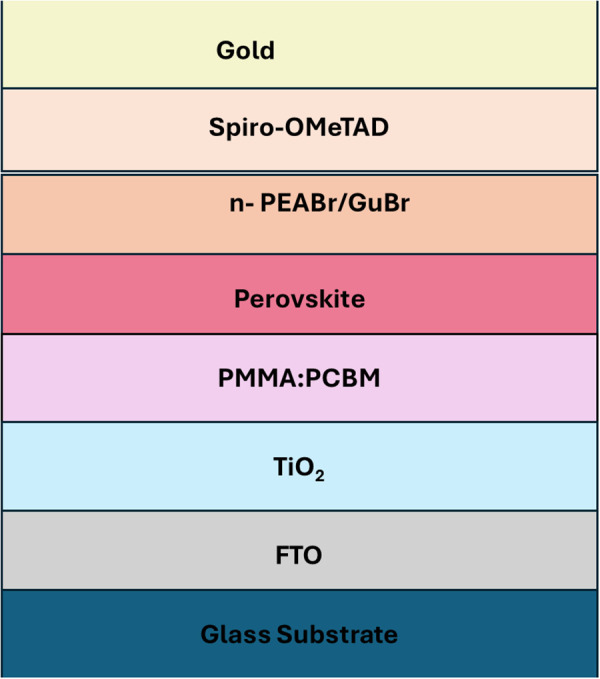
PSC structure.

**Fig 2:**
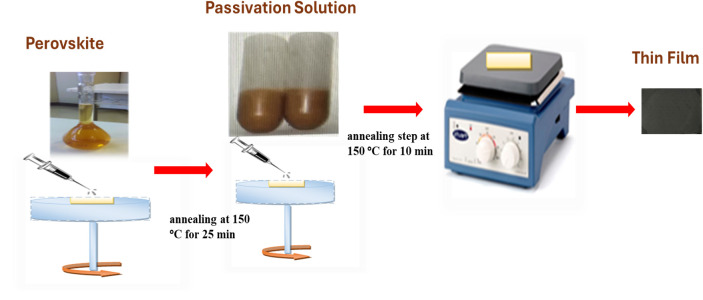
The introduction of PEABr, GuaBr, and their blend onto the surface.

Schematic illustrations of the device deposition procedures and structure are depicted in Figs 1 and 2. Before conducting the measurements, the light intensity was adjusted using a certified Fraunhofer CalLab reference cell. The J-V characteristic was recorded with a scan rate of 50 mV/s and a voltage step of 0.01 V, with the cells positioned in a specially designed measurement fixture and exposed to a flow of nitrogen gas. For the forward scan, a voltage range of −0.1 V to 1.25 V was used, while the reverse scan employed a range from 1.25 V to −0.1 V. The characterization was carried out without any prior preconditioning protocol. It is important to highlight that the short-circuit current density (Jsc) shows a slight increase as the ratio of Phenylethylammonium is raised. Specifically, the cell with the 1P-1G passivation layer exhibits a lower Jsc compared to the cell with the 1P-2G passivation layer. On the other hand, the cell with the 2P-1G passivation layer achieves the highest Jsc. This observed trend can be attributed to the significant impact of Phenylethylammonium passivation. Additionally, the higher Jsc and open-circuit voltage (Voc) in some cases might be due to reduced charge recombination, which suggests improved efficiency in charge carrier management.

Among the various compositions of cations with combined spacers, all showed comparable current-voltage (J-V) characteristics. However, the PSC with the 2P-1G passivation layer displayed superior stability over time. Therefore, this composition was selected as optimal for further characterization. The experimental procedure is shown in Fig 2. A sufficient number of devices were fabricated under each condition to ensure the reliability of the reported results. The device performance was evaluated based on multiple independent samples, and the averaged values were used to draw conclusions. In terms of reproducibility, the devices exhibited consistent performance with only small variations in the key photovoltaic parameters (such as Jsc, Voc, FF, and PCE). This indicates good reproducibility of the fabrication process. The consistency can be attributed to the robustness of the processing method, particularly the rapid thermal treatment, which minimizes variability associated with prolonged annealing. Statistical data (e.g., average values and standard deviations) have been included to further support the reproducibility of the results.

### 2.2 Characterization

X-ray diffraction analyses were conducted utilizing theta-2theta geometry with Cu Kα radiation (wavelength: 1.56 Å) employing an X-ray diffractometer (model Explorer, manufactured by GNR Analytical Instruments in Novara, Italy. For the characterization of thin-film morphology, a high-resolution field emission scanning electron microscope (FE-SEM) JSM-7800F Prime (JEOL Ltd., Tokyo, Japan) equipped with an in-lens Schottky plus field emission electron gun was employed. The SEM utilized a thin conductive layer of Pt, approximately 20 Å thick, deposited on the films prior to imaging. The surface morphology of the samples was examined using a FEI Verios SEM with an acceleration voltage of 2 kV and a probe current of 25 pA. UV-Vis spectra were obtained with a UV/VIS spectrometer (Perkin-Elmer Lambda 35, manufactured by PerkinElmer Instruments in Shelton, CT, USA), while photoluminescent (PL) spectra were collected using a fluorescence spectrophotometer (Perkin-Elmer LS55). The cross-sectional sample of the perovskite solar cell was prepared using Helios NanoLab 600 Focused Ion Beam (FIB) milling. The microstructure of the perovskite device was examined with a JEOL 2100F transmission electron microscope (TEM) operating at 200 keV, while atomic force microscopy (AFM) topographic analysis was performed using a Bruker Icon scanning probe microscope.

## 3 Result and discussion

**Fig 3:**
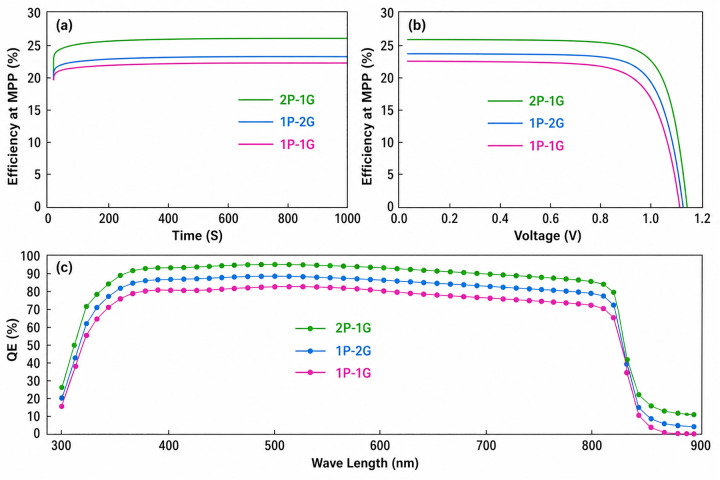
(a) Efficiency per time, (b) The J-V of 1P-1G, 2P-1G and 1P-2G, (c) The QE curves of simulated perovskite films of 1P-1G, 2P-1G and 1P-2G.

Fig 3 illustrates the distributions of photovoltaic characteristics for the devices that were fabricated. It is evident from the data that the device incorporating a 2P-1G passivation layer achieved an increase in PCE compared to both 1P-1G and 1P-2G cells, reaching a peak efficiency of 25.11%for the top-performing cell. This overall enhancement in efficiency across all passivated devices can be attributed to improvements in both fill factor (FF) and open-circuit voltage (${\text{V}}_{OC}$) observed in these samples. The superior performance of the 2P-1G dual-cation passivation strategy arises from the complementary mechanisms of n-phenylethylammonium bromide (PEABr) and guanidinium bromide (GuaBr). PEABr primarily contributes to interfacial passivation: its aromatic phenyl group forms π−π stacking interactions and hydrogen bonds with undercoordinated iodide species, effectively reducing surface trap states, suppressing non-radiative recombination, and enhancing charge extraction. GuaBr, in contrast, is small enough to partially diffuse into the perovskite bulk and localize at grain boundaries, where it passivates deep defects, reduces ion migration, and improves grain boundary stability. Together, these effects create a synergistic balance: PEABr improves electronic coupling and hydrophobic stability at the surface, while GuaBr mitigates bulk defects and stabilizes crystal growth. This dual-action mechanism explains why the 2P-1G configuration achieved higher efficiency (PCE 25.11%) and superior stability compared to single-cation passivation, which lacks either strong interfacial passivation (GuaBr alone) or bulk defect suppression (PEABr alone). Specifically, the application of the passivation layer led to increased ${\text{V}}_{OC}$ values across all conditions when compared to the 1P-1G sample, within the highest average ${\text{V}}_{OC}$ of 1.21 V recorded for the 1P-1G cell. Notably, the 2P-1G configuration exhibited an average ${\text{V}}_{OC}$ of 1.23 V, surpassing that of other cell types tested. The superior performance of the 2P-1G (PEABr:GuaBr) configuration can be attributed to a synergistic mechanism between the two spacer cations, with phenylethylammonium (PEABr) playing the dominant role. The excess of PEABr enhances surface passivation by forming strong hydrogen bonds with undercoordinated iodide ions and reducing non-radiative recombination at the perovskite/HTL interface, thereby improving charge extraction and short-circuit current density (Jsc). At the same time, the aromatic phenyl group facilitates π−π stacking, which contributes to improved structural rigidity and interfacial stability. Guanidinium (GuaBr), on the other hand, partially diffuses into the perovskite bulk and localizes at grain boundaries, where it reduces trap density and ion migration. Thus, the combination of bulk passivation from GuaBr and superior interfacial passivation from PEABr leads to enhanced long-term stability. The observed improvement in Jsc and device durability in the 2P-1G sample can therefore be explained as a cooperative effect, where PEABr dominates surface/interface quality while GuaBr complements by passivating grain boundaries. This increase in ${\text{V}}_{OC}$ across the passivated samples suggests a reduction in nonradiative recombination, a topic to be explored further. In Fig 3c, it can be observed that the addition of the passivation layer at the HTL/perovskite interface resulted in a slight improvement in FF for all treated conditions. Unlike certain other passivation methods such as PMMA:PCBM, which often sacrifice FF for voltage gains, the methods employed here maintained or slightly enhanced FF while improving${\text{V}}_{OC}$. Furthermore, the device with 2P-1G passivation displayed the highest short-circuit current density (${\text{J}}_{SC}$), which contributed significantly to its superior PCE compared to both 1P-1G and 1P-2G passivated devices. The underlying reasons for the increased ${\text{J}}_{SC}$ will be discussed in detail later.

**Fig 4:**
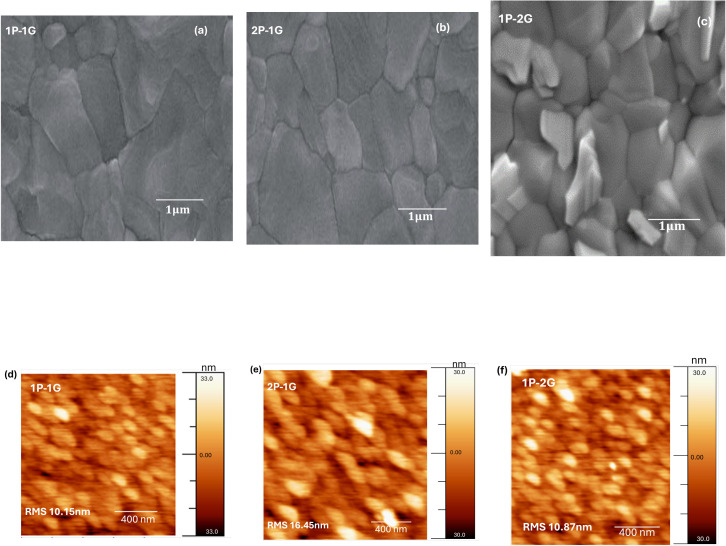
(a-c) SEM images and (d–f) AFM images of the perovskite films.

Furthermore, Fig 3 displays the statistical distribution of device performance, showing enhancements primarily due to increased open-circuit voltage $\left({\text{V}}_{oc}\right)$ and fill factor (FF). Each device exhibits minimal photocurrent hysteresis at a scan rate of 55 ${\text{mV s}}^{-1}$. Hysteresis is indeed a well-documented challenge in perovskite solar cells due to charge trapping/detrapping and ion migration at interfaces. In our study, J–V measurements were conducted without preconditioning, including both forward and reverse scans, to capture potential hysteresis effects. The results (Fig 3b) revealed minimal hysteresis across all devices, indicating that the applied passivation strategies effectively suppressed interfacial trap states. Among the three passivation conditions, the 2P-1G configuration exhibited the least hysteresis, consistent with its higher Voc and Jsc. This behavior can be attributed to the synergistic action of PEABr and GuaBr: PEABr reduces non-radiative recombination and interfacial trap density through strong surface passivation, while GuaBr localizes at grain boundaries, mitigating ion migration. The reduced hysteresis observed in 2P-1G devices therefore confirms that dual-cation passivation not only enhances efficiency and stability but also significantly improves charge carrier dynamics by minimizing trap-mediated charge accumulation. The light-soaking stability of the devices was evaluated under continuous 1-sunlight illumination in an inert atmosphere (nitrogen gas), with the cells’ voltage fixed at the initial voltage at maximum power point (VMPP). As depicted in Fig 4a, the efficiencies at MPP remained stable after 1100 seconds for all conditions. Additionally, a shelf-life stability assessment testing was performed on different uncovered devices. exposed to ambient atmosphere with approximately 80% relative humidity over 25 days. The results indicated that the 2P-1G cell retained approximately 80% of its initial efficiency, demonstrating superior stability compared to other conditions. All stability tests in this study were conducted on unencapsulated devices to directly assess the intrinsic effects of passivation. Under shelf-life conditions (80% RH) and continuous light-soaking in an inert atmosphere, the 2P-1G device retained 80% of its initial efficiency after 25 days, which we attribute to the strong hydrophobicity and defect passivation imparted by the dual-cation layer. While these results demonstrate significant improvement compared to single-cation passivation, we acknowledge that real-world outdoor environments impose more stringent stress factors, including fluctuating temperature, higher humidity, and prolonged illumination. Given the demonstrated hydrophobicity of phenylethylammonium and the grain boundary passivation of guanidinium, we anticipate that the 2P-1G device would maintain superior stability relative to non-passivated or single-passivated devices under operational conditions. However, further studies involving encapsulated modules under accelerated aging protocols (e.g., damp-heat, thermal cycling, and outdoor field testing) will be essential to fully validate the long-term operational stability of this passivation strategy. Moreover, a light-soaking stability test was performed over 50 hours, during which J–V curves were continuously monitored at hourly intervals. Fig 4b illustrates the comparative J–V performance of leading PSCs, namely 1P-1G,2P-1G, and 1P-2G. It is evident that the 2P-1G cells exhibit a notable enhancement in power conversion efficiency (PCE), increasing from 23.2% to 25.11% compared to the as-prepared cells.

This improvement primarily results from a significant increase in short-circuit current density (${\text{J}}_{SC}$) and a slight improvement in fill factor (FF). Notably, the highest PCE of 25.11% was achieved with the 2P-1G configuration. The enhanced PCE can be attributed to the increase in${\text{V}}_{OC}$ from 1.21 V to 1.23 V and an FF of 85.7%, despite a slight decrease in ${\text{J}}_{SC}$ to ${\text{24.96mAcm}}^{-2}$. Statistical analysis of PCE distribution confirms the consistency of these results, underscoring that the dual surface passivation using guanidinium and phenylethylammonium spacer cations effectively enhances PSC performance. The incident photon-to-current efficiency (IPCE) was evaluated using the QEX10 Solar Cell Quantum Efficiency Measurement System. In Fig 4c, the IPCE curves of perovskite solar cells 1P-1G, 2P-1G and 1P-2G are illustrated. These curves exhibit a gradual decrease from the 300–400 nm range, tapering off towards the absorption edge at approximately 820 nm. Notably, the device 2P-1G displayed superior quantum efficacy, indicating higher quality. Efficient transfer of electrons and holes was observed within the high-energy range of visible light, while optimal device interfaces were crucial in the low-energy spectrum (600–800 nm). The decline in efficiency of these solar cells can be attributed to spectral mismatch, where the input energy of photons does not align with the solar cell’s band gap. Photons with lower energy than the bandgap cannot be absorbed, leading to their excess energy being converted into the kinetic energy of electron-hole pairs, which is subsequently dissipated as heat. This outcome demonstrates a significant suppression of non-radiative losses in the perovskite layer through dual surface passivation utilizing phenylethylammonium and guanidinium spacer cations. If we could completely eliminate non-radiative recombination in the perovskite layer, whether at the surface or the interface, we could potentially raise the ${\text{V}}_{OC}$ closer to its limit of 1.25 V. This improvement could potentially increase the PCE of PSCs over 25%. J_SC_ integrated from EQE data in Fig 4c, are listed in Table 1.

**Table 1:** J_SC_ integrated from EQE data.

Device	Short-circuit current Density (Jsc) (*mA*/*cm*^2^)
1P-1G	24.73
2P-1G	24.96
1P-2G	23.89

**Fig 5:**
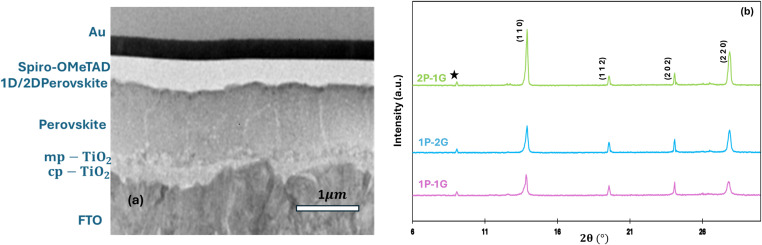
(a) BR-TEM image of the PSC device at perovskite/HTL interface, (b) XRD of 1P-1G, 2P-1G and 1P-2G.

**Fig 6:**
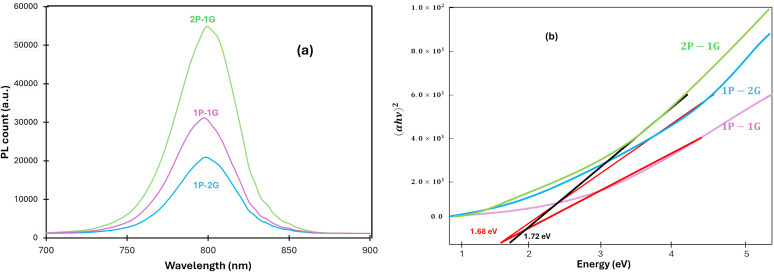
(a)The normalized PL spectra for samples 1P-1G,2P-1G and 1P-2G, (b) Tauc plot.

To understand why the 2P-1G sample is more efficient, a detailed analysis of its properties was conducted. Initially, the morphology of surface of the samples was examined using scanning electron microscopy (SEM) (Fig 5a–5c). Upon application of the PEABr passivation layer on the perovskite absorber, a slight increase in grain size was observed, likely due to the Ostwald ripening process facilitated by the partial dissolution of perovskite crystals in an IPA solution containing bromide. [54,58] The grain size remained relatively unchanged in films with 1P-1G and 2P-1G configurations. Additionally, atomic force microscopy (AFM) analysis of surface roughness revealed the most even surface for the 2P-1G sample, with a root-mean-square (RMS) roughness of 7.47 nm, compared to 10.15 nm for 1P-1G, 10.87 nm for 1P-1G, and the reference film (Fig 5d-5f). A smoother perovskite layer surface can mitigate nonradiative recombination and facilitate improved charge extraction at the interface by enhancing contact with the transport layer. [53,65,66] At low magnification, cross-sectional bright-field transmission electron microscopy (BF-TEM) images revealed a thin passivation layer that was not prominently visible at the HTL/perovskite interface. However, high-resolution TEM clearly revealed a uniform, thin passivation layer at the interface, with measured lattice spacings of 7.018 Å and 3.789 Å, corresponding to the 1D/2D structure of the passivation layer and the structure of the perovskite bulk, in their particular cases (Fig 6a), consistent with previous studies [52]. It is noted that distinguishing between 1D and 2D perovskite structures in TEM images can be challenging due to their similar lattice spacings [67]. The passivation layer thickness was estimated to be approximately 20 nm based on BF-TEM images. X-ray diffraction was employed to characterize the crystallographic changes induced by different passivation layers (Fig 6b). The main peaks observed in all films correspond to the perovskite structure. Additional peaks at 3.2° and 9.9° in the 1P-1G and 2P-1G samples indicate the presence of 2D perovskite layers. Meanwhile, a peak at 9.9° in the 1P-2G sample suggests the formation of 1D perovskite. The X-ray diffraction (XRD) patterns show that the intensity of peaks related to 1D/2D structures is minimal compared to the predominant peaks from the perovskite structure [36,67,74]. We acknowledge that the XRD signals corresponding to 1D/2D perovskite phases appear with relatively low intensity compared to the dominant 3D perovskite peaks, which can make interpretation challenging. However, our structural assignment is supported by complementary characterizations. High-resolution TEM (Fig 5a) clearly revealed a thin and continuous passivation layer at the perovskite/HTL interface, with lattice spacings of 7.018 Å and 3.789 Å consistent with 1D/2D layered structures. Additionally, steady-state and time-resolved photoluminescence (PL and TRPL, Fig 6a, Table 2) confirmed suppressed non-radiative recombination and extended carrier lifetimes, both characteristic signatures of effective 1D/2D surface passivation. AFM results further showed smoother surfaces in the 2P-1G films, consistent with uniform low-dimensional capping. While GIWAXS was not performed in this study, the convergence of evidence from XRD, TEM, PL, and AFM provides strong confidence in the presence of low-dimensional perovskite phases at the interface. From these results, the highest amount of 2D perovskite is observed in the P-passivated film, followed by the 2P-1G film. The 2P-1G film, which includes both P and G passivation agents, contains a mixture of 2D structures and a small amount of 1D structures. Previous studies using conventional X-ray diffraction did not detect specific peaks for films with low concentrations of guanidinium cation [58,67,68]. SEM cross-sectional images for the other PSCs have now been included to provide a comprehensive comparison of the device architecture under different processing conditions. These cross-sectional images confirm the formation of well-defined and continuous layers across all devices, including the perovskite absorber, transport layers, and electrodes. No significant differences in layer thickness or interfacial contact are observed, indicating that the fabrication process yields consistent device structures. Minor variations in morphology may be present, particularly in terms of film compactness and grain connectivity, which can influence charge transport and recombination. However, overall, the cross-sectional SEM images demonstrate that all PSCs exhibit good layer uniformity and intimate interfacial contact, supporting the observed device performance.

**Table 2:** Life time for different samples.

Sample	Time (ns)
1P-1G	296
2P-1G	315
1P-2G	276

Steady-state photoluminescence (SS-PL) spectroscopy was employed to analyze the charge recombination behavior of perovskite films on bare glass both before and after implementing surface passivation. The results revealed the highest PL intensity across all samples (Fig 6a), indicating that dual cations effectively reduce nonradiative charge recombination. Time-resolved PL (TRPL) measurements confirmed these findings, showing reduced decay in treated on the surface perovskite samples (Table 2). Lifetime calculations using a biexponential model yielded values of 296 ns for 1P-1G, 315 ns for 2P-1G, and 276 ns for 1P-2G, consistent with trends observed in ${\text{V}}_{OC}$ and highlighting the passivation layers’ efficacy in suppressing nonradiative carrier recombination.To further explore optical performance, ultraviolet–visible–near infrared (UV–vis–NIR) spectroscopy was employed. Tauc plot (Fig 6b) exhibited significant differences among samples, indicating the impact of passivation layers on optical properties. The optical bandgaps were determined via Tauc plot analysis, revealing variations among samples. Specifically, the minimum bandgap about of 1.68 eV was calculated for 1P-1G and 1P-2G samples, while the 2P-1G sample exhibited a slightly higher bandgap of 1.72 eV. These results underscore the influence of passivation strategies on both photoluminescence dynamics and optical properties of perovskite films.

Photoluminescence (PL) is a valuable technique for assessing disorder within a system and determining the purity and crystalline quality of semiconductors. The photoluminescence spectra (PL) for all films excited by a 450 nm laser are presented. Each sample displays a single peak within the range of 805–814 nm. The steady-state PL spectra reveal that the absorbance and PL peaks of perovskite films are consistently shiftedfor 2P-1G.

**Fig 7:**
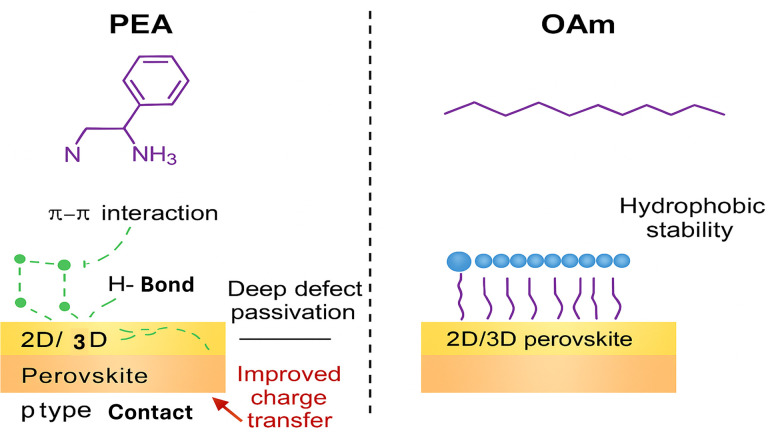
Comparative passivation mechanisms of phenylethylammonium (PEA) and octylammonium (OAm) at the perovskite/HTL interface.

The 2P-1G sample, which exhibits a slightly higher optical bandgap of 1.72 eV compared to ∼1.68 eV for the other samples, shows a corresponding blue-shift in the absorption edge, indicating that photon absorption is shifted toward shorter wavelengths. This behavior is consistent with the fundamental relationship between bandgap and absorption, where an increase in bandgap results in reduced absorption in the low-energy (long-wavelength) region. As observed from the UV–Vis and EQE spectra (Figs 3c and 7b), the absorption onset of the 2P-1G device occurs at a shorter wavelength (∼720–750 nm), confirming the widened bandgap.

Despite this slight reduction in near-band-edge absorption, the 2P-1G sample maintains high overall absorption in the visible region (300–750 nm), which is attributed to improved film quality and reduced defect density. The enhanced absorption efficiency is further supported by the higher EQE response across the visible spectrum, indicating more effective photon harvesting and charge generation. Importantly, the improved photovoltaic performance (PCE = 25.11%) is not limited by the increased bandgap, as the reduction in non-radiative recombination and improved charge extraction compensate for the slight loss in long-wavelength absorption. This behavior is consistent with recent reports on higher-bandgap perovskites. For instance, Qi Jiang et al. demonstrated that surface-passivated perovskites with slightly increased bandgaps (∼1.7–1.75 eV) can achieve PCEs above 23%, owing to suppressed non-radiative recombination and enhanced open-circuit voltage. Similarly, Naeimeh Mozaffari reported PCE ≈23.13% using binary passivation strategies, where improved interfacial quality outweighed minor absorption losses. These studies highlight that bandgap widening, when accompanied by effective passivation, can lead to higher device efficiency through improved voltage and carrier dynamics rather than purely enhanced absorption. Our study underscores the effectiveness of passivation in the 2P-1G configuration, highlighting the crucial role played by PEABr in enhancing the performance of perovskite solar cells (PSCs). PEABr’s composition contributes significantly to this improvement, leveraging its unique electronic and optical properties. Specifically, PEABr aids in reducing nonradiative recombination processes within the perovskite layer, thereby enhancing charge carrier lifetime and overall photovoltaic efficiency. This passivation effect is crucial as it helps to mitigate losses in open-circuit voltage (${\text{V}}_{OC}$) and fill factor (FF), ultimately leading to higher power conversion efficiencies (PCEs). The electronic structure of PEABr facilitates improved charge transport properties and better energy level alignment at the interfaces, which is essential for efficient charge extraction and collection within the solar cell device. Phenylethylammonium bromide (PEABr) plays a dual role in enhancing both the electronic and optical properties of perovskite solar cells in the 2P-1G configuration. Electronically, the phenyl group in PEABr forms strong hydrogen bonds with undercoordinated iodide ions at the perovskite surface, effectively reducing trap density and suppressing non-radiative recombination. Its conjugated aromatic structure also facilitates improved electronic coupling and energy level alignment, which enhances charge extraction and reduces voltage losses. Optically, PEABr contributes to a smoother and more uniform perovskite surface, as confirmed by AFM, which minimizes scattering losses and promotes more efficient light absorption. Additionally, π−π stacking interactions from the aromatic rings increase structural rigidity at the interface, further improving stability. Compared to octylammonium, which mainly offers hydrophobic protection but introduces an insulating barrier due to its long alkyl chain, PEABr provides a better balance between hydrophobicity, defect passivation, and charge transport. This explains why PEABr-based passivation in the 2P-1G system outperforms octylammonium, achieving higher PCE (>25%vs~23%) and superior long-term stability. Furthermore, PEABr’s optical properties contribute to enhanced light absorption and utilization within the perovskite layer, thereby maximizing photon-to-electron conversion efficiency. Overall, our findings underscore PEABr as a pivotal component in optimizing the performance of PSCs, highlighting its dual role in enhancing both electronic and optical characteristics critical for achieving high-efficiency photovoltaic devices. The innovative aspect of this manuscript lies in the application of phenylethylammonium in conjunction with guanidinium spacer cations for dual surface passivation of perovskite layers. Our study demonstrates that phenylethylammonium alone provides superior passivation compared to previous methods. We have achieved a Power Conversion Efficiency (PCE) exceeding 25%, surpassing the 23% PCE achieved by Mozaffari using octylammonium. Replacing octylammonium with phenylethylammonium provides distinct mechanistic advantages for passivation in perovskite solar cells. As shown in Fig 7, the aromatic phenyl group of phenylethylammonium introduces π−π stacking interactions and stronger hydrogen bonding with undercoordinated iodide species, leading to more effective defect passivation and suppression of non-radiative recombination. Additionally, its conjugated π−system enhances electronic coupling and charge transfer at the interface, while maintaining sufficient hydrophobicity to protect against moisture. In contrast, long-chain alkylammonium cations such as octylammonium primarily provide hydrophobic stability but act as insulating barriers, which can hinder charge transport due to their bulky, flexible chains. This balance of structural rigidity, defect passivation, and efficient carrier extraction explains why phenylethylammonium outperforms octylammonium in both device efficiency and long-term stability. The selection of phenylethylammonium is justified by its exceptional passivation capabilities, coupled with its advantageous optical and electrical properties. These attributes contribute to improved efficiency in perovskite solar cells, highlighting the effectiveness of phenylethylammonium as a passivation material.

The enhanced photovoltaic performance of the dual-cation–passivated devices can be attributed to a synergistic reduction in both interfacial and bulk defect densities, which directly suppresses non-radiative recombination pathways. In particular, the increase in open-circuit voltage ($V_{OC}$) from ∼1.21 V to 1.23 V for the 2P-1G device indicates improved quasi-Fermi level splitting due to effective passivation of trap states at the perovskite/HTL interface. Phenylethylammonium bromide (PEABr) primarily contributes to surface passivation by forming a low-dimensional (1D/2D) perovskite capping layer that reduces undercoordinated Pb^2+^ defects and acts as a physical barrier against charge recombination. In contrast, guanidinium bromide (GuaBr), owing to its smaller ionic size, partially penetrates the perovskite lattice and passivates grain boundaries through hydrogen bonding with halide ions, thereby reducing deep-level trap states. This complementary passivation mechanism leads to a significant decrease in Shockley–Read–Hall recombination, which is further supported by the prolonged carrier lifetime observed in time-resolved photoluminescence measurements (315 ns for 2P-1G).

In addition to $V_{OC}$ improvement, the increase in short-circuit current density ($J_{SC}$) to 24.96 mA cm^−2^ is associated with enhanced charge collection efficiency and improved interfacial charge transport. The reduced trap density extends carrier diffusion lengths, allowing photogenerated carriers to reach the electrodes with minimal recombination losses. Morphological analysis reveals a smoother and more uniform perovskite surface (RMS roughness of 7.47 nm), which improves physical contact with the hole transport layer and facilitates efficient charge extraction. Furthermore, incident photon-to-current efficiency (IPCE) measurements indicate superior spectral response across the visible region, suggesting that electrical improvements outweigh the slight increase in bandgap (1.72 eV) observed for the 2P-1G sample. The marginal bandgap widening can be attributed to the formation of low-dimensional perovskite phases and reduced band tailing, which minimizes sub-bandgap recombination losses.

The fill factor (FF) enhancement to ∼85.7% further confirms improved charge transport dynamics and reduced series resistance within the device. Unlike conventional passivation strategies that often compromise FF, the dual-cation approach maintains efficient carrier extraction by optimizing energy level alignment at the perovskite/HTL interface and minimizing charge accumulation. Structural characterization using X-ray diffraction reveals the coexistence of 2D and minor 1D perovskite phases, forming a mixed-dimensional heterostructure that combines the superior charge transport properties of 3D perovskites with the defect passivation and environmental stability of low-dimensional layers. This architecture effectively reduces ion migration and enhances interfacial stability.

Photoluminescence analysis provides direct evidence of suppressed non-radiative recombination, as indicated by increased PL intensity and extended carrier lifetimes. These findings confirm that the dual-passivation strategy significantly improves the optoelectronic quality of the perovskite films. Moreover, the enhanced environmental stability, with ∼80% efficiency retention after prolonged exposure to high humidity conditions, is attributed to the hydrophobic nature of the PEABr layer and the stabilization of iodide ions by guanidinium through hydrogen bonding. This combination effectively inhibits moisture ingress and ion migration, which are primary degradation pathways in perovskite solar cells.

Overall, the superior performance of the 2P-1G configuration arises from an optimal balance between surface and bulk passivation. A higher proportion of PEABr ensures effective surface defect suppression and moisture resistance, while an appropriate amount of GuaBr enables efficient bulk defect passivation without disrupting the perovskite lattice. This dual-cation strategy therefore simultaneously enhances charge carrier dynamics, reduces recombination losses, and improves structural stability, ultimately leading to high power conversion efficiency exceeding 25% and improved device durability.

**Table 3:** Comparison of single- and dual-cation passivation strategies in PSCs.

Study	Cations Used	Structure Formed	$V_{OC}$ (V)	PCE (%)
This work (Dual)	PEABr + GuaBr	Mixed 1D/2D–3D	1.23	25.11
Mozaffari et al. [72] (Dual)	GuaBr + Octylamm.	1D/2D–3D	1.21	23.13
Jiang et al. (Single) [71]	PEA^+^	2D/3D interface	∼1.19	∼23.3
Chavan et al. [68] (Single)	GuaI / GuaBr	Bulk-modified 3D	∼1.17	∼20.0
PEAI engineering (Single)	PEAI	2D capping layer	—	∼22
GAI + diammonium (Dual)	GAI + diammonium	2D/3D heterostructure	—	>23
Methoxy-PEAI (Single)	Modified PEAI	2D layer	1.14	19.15

A comparison with previously reported passivation strategies clearly highlights the advantages of dual-cation engineering over single-cation approaches. Single-cation systems such as phenylethylammonium (PEA^+^) primarily improve surface defect passivation by forming a 2D perovskite capping layer, which enhances open-circuit voltage ($V_{OC}$) through suppression of interfacial recombination. However, their effect is largely confined to the surface, limiting their ability to address bulk defects and grain boundary recombination. In contrast, guanidinium-based passivation exhibits the opposite behavior, where smaller cations can penetrate into the perovskite lattice and effectively passivate bulk and grain boundary defects, but provide weaker surface protection (Table 4).

**Table 4:** Key Findings for Table 3.

Study	Key Findings
This work	Synergistic surface & bulk passivation; reduced recombination; improved stability
Mozaffari et al.	Enhanced hydrophobicity; improved film uniformity and $V_{OC}$
Jiang et al.	Effective surface defect passivation; limited bulk passivation
Chavan et al.	Bulk defect healing; improved grain boundary passivation
PEAI engineering	Reduced interfacial recombination; improved energy alignment
GAI + diammonium	Simultaneous buried and surface passivation; enhanced stability
Methoxy-PEAI	Improved stability; moderate efficiency enhancement

Dual-cation strategies, such as the combination of guanidinium with octylammonium reported by Mozaffari et al. [61], demonstrate improved device performance (PCE ≈23.13%) due to the formation of mixed-dimensional (1D/2D–3D) structures that simultaneously enhance surface hydrophobicity and reduce recombination losses. More recent studies further confirm that combining different organic cations enables simultaneous passivation of both exposed and buried interfaces, leading to improved charge transport and stability.

**Fig 8:**
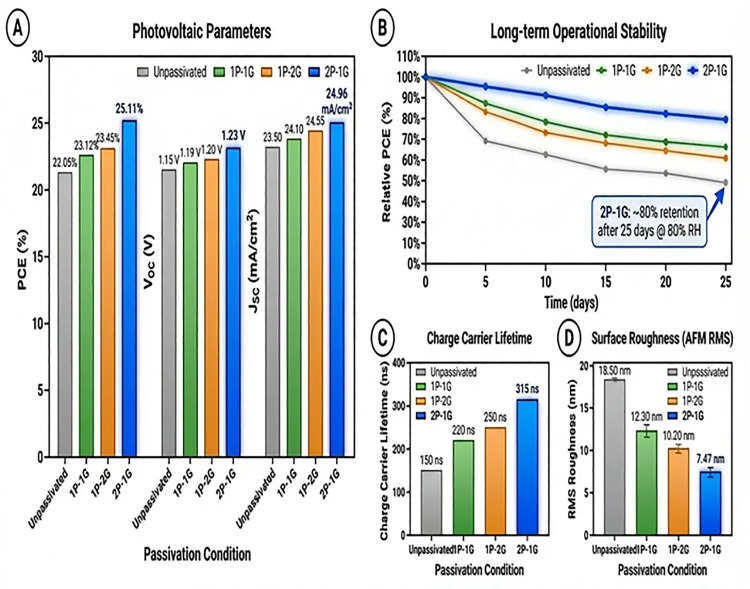
2P-1G dual-cation passivation yields highest efficiency (25.11% PCE), prolonged stability (∼80% after 25 days @ 80% RH), extended carrier lifetime (315 ns), and smoothest morphology (7.47 nm rms).

In this context, the present work achieves a superior PCE of 25.11% by employing a dual-cation system (PEABr + GuaBr), which optimally balances surface and bulk passivation. Unlike earlier dual-cation systems, replacing long alkyl chains (e.g., octylammonium) with phenylethylammonium introduces improved electronic coupling and charge transport, while maintaining hydrophobic protection. This results in enhanced carrier lifetime, reduced trap density, and improved energy level alignment. Consequently, the dual-passivation strategy presented here outperforms both single-cation and previously reported binary systems, demonstrating that rational selection of complementary cations is critical for maximizing both efficiency and stability in perovskite solar cells. Fig 8 comprehensively summarizes the impact of different passivation strategies on the performance and stability of perovskite solar cells. As shown in panel (A), the dual-cation 2P-1G device exhibits the highest photovoltaic performance, achieving a PCE of 25.11%, along with an enhanced open-circuit voltage ($V_{OC}$) of 1.23 V and a high short-circuit current density ($J_{SC}$) of 24.96 mA cm^−2^. These values clearly outperform both the single-cation and unpassivated devices.

Panel (B) illustrates the long-term operational stability under ambient conditions (80% relative humidity). The 2P-1G device retains approximately 80% of its initial efficiency after 25 days, demonstrating significantly improved stability compared to the other samples, which show more pronounced degradation over time.

Furthermore, panel (C) reveals a substantial increase in charge carrier lifetime for the 2P-1G sample, reaching 315 ns. This enhancement indicates effective suppression of non-radiative recombination, which is a key factor contributing to the improved device performance.

This observation is supported by the morphological analysis shown in panel (D). Atomic force microscopy (AFM) results indicate that the 2P-1G film exhibits the lowest surface roughness (RMS = 7.47 nm), confirming the formation of a smoother and more uniform perovskite surface compared to the other passivation conditions.

## 4 Conclusion

To summarize, we employed structural manipulation for efficient passivation the perovskite/HTL interface using dual spacer cations, namely n-PEABr and GuaBr. The binary cation passivation layer resulted in a 50 mV increase in ${\text{V}}_{OC}$ in contrast to the control device, achieving a maximum efficiency of 25.11% and outperforming devices with single-cation PEABr passivation. Our stability tests under ambient conditions with 80% relative humidity identified the 2P-1G sample regarded as highly stable, attributed to its high contact angle that repels humidity and low imperfection density. In light-soaking stability tests, the 1P-1g device exhibited reduced nonradiative recombination centers, validated by photoluminescence measurements, and improved interaction with the adjacent HTL, facilitating better charge extraction. X-ray diffraction analysis revealed two peaks indicative of 2D perovskite in both 1P-1G and 2P-1G samples, with a minimal 1D property peak in the P-passivated sample. Consistent with prior studies using phenylethylammonium cations for surface passivation, we hypothesize that these ions typically accumulate at perovskite grain boundaries, forming hydrogen bonds with undercoordinated iodine species to inhibit iodide ion migration. High-resolution TEM imaging verified the presence of a uniform, continuous 1D/2D passivation coating predominantly originating from PEABr atop the 3D perovskite layer in the mixed-cation passivation sample. Furthermore, space-charge-limited current measurements indicated a threefold increased trap-dense limit voltage in the 1P-1G sample compared to the 2P-1G sample, suggesting fewer trapped states in the passivated cell. In conclusion, the enhanced photovoltaic effectiveness of the 2P-1G passivated cell can be credited to reduced recombination trap states and improved charge collection efficiency. Exploring alternative dual spacer cations for surface passivation holds promise for further enhancing cell performance and stability in future studies.

## References

[1] Li F, Deng X, Qi F, Li Z, Liu D, Shen D. J Am Chem Soc. 2020;142:20134. 33190487 10.1021/jacs.0c09845

[2] Jeong MJ, Yeom KM, Kim SJ, Jung EH, Noh JH. Energy Environ Sci. Energy Environ Sci. 2021;14:2419.

[3] Wang P, Li R, Chen B, Hou F, Zhang J, Zhao Y. Adv Mater. 2020;32:1905766. 10.1002/adma.201905766 31899829

[4] Jeon NJ, Na H, Jung EH, Yang T, Lee YG, Kim G. Nat Energy. 2018.

[5] N R E L. Best research-cell efficiency chart. 2021. https://www.nrel.gov/pv/cell-efficiency.html

[6] Kojima A, Teshima K, Shirai Y, Miyasaka T. J Am Chem Soc. 2009;131:6050. 19366264 10.1021/ja809598r

[7] Zhu P, Chen C, Dai J, Zhang Y, Mao R, Chen S, et al. Toward the Commercialization of Perovskite Solar Modules. Adv Mater. 2024;36(15):e2307357. doi: 10.1002/adma.202307357 38214179

[8] Chauhan AK, Kumar P, Sharma SN. Perovskite solar cells: Past, present, and future. Photovoltaics Beyond Silicon. 2024. p. 113–63. 10.1016/B978-0-323-90188-8.00015-4

[9] Commercial perovskites imminent. PV Magazine. 2023. https://www.pv-magazine.com/2023/10/31/commercial-perovskites-imminent/

[10] Xia J, Liang C, Gu H, Mei S, Li S, Zhang N, et al. Surface passivation toward efficient and stable perovskite solar cells. Energy & Environmental Materials. 2023;6:e12296.

[11] Hui W, Chao L, Lu H, Xia F, Wei Q, Su Z. Science. 2021;371:1359. 10.1126/science.abf7652 33766883

[12] Wang R, Huang T, Xue J, Tong J, Zhu K, Yang Y. Nature Photonics. 2021;15:411.

[13] NREL. Best Research-Cell Efficiency Chart. https://www.nrel.gov/pv/cell-efficiency.html

[14] Green MA, Dunlop ED, Yoshita M, Kopidakis N, Bothe K, Siefer G. Solar cell efficiency tables (Version 64). Progress in Photovoltaics: Research and Applications. 2024;32:425–41.

[15] P V T I M E. 871 million yuan! Perovskite pilot line to be launched in Anhui province of China. 2024. Accessed 2024 July 12.

[16] Chen J, He D, Park NG. Sol RRL. 2022;6:2100767.

[17] Zhu L, Zhang X, Li M, Shang X, Lei K, Zhang B. Adv Energy Mater. Adv Energy Mater. 2021;11:2100529.

[18] Bu T, Li J, Li H, Tian C, Su J, Tong G. Science. 2021;372:1327. 10.1126/science.abh1035 34140385

[19] Xie J, Yan K, Zhu H, Li G, Wang H, Zhu H. Sci Bull. 2020;65:1726. 10.1016/j.scib.2020.05.031 36659245

[20] Xie L, Vashishtha P, Koh TM, Harikesh PC, Jamaludin NF, Bruno A. Advanced Materials. 2020;32:2003296. 10.1002/adma.202003296 32856340

[21] Marchioro A, Teuscher J, Friedrich D, Kunst M, Van De Krol R, Moehl T. Nat photonics. Nat Photonics. 2014;8:250.

[22] Chong WK, Giovanni D, Sum TC. Halide Perovskites Photovoltaics, Light Emitting Devices, and Beyond. Weinheim: Wiley-VCH. 2018.

[23] Rajagopal A, Yao K, Jen AKY. Adv Mater. Adv Mater. 2018;30:1800455. 10.1002/adma.201800455 29883006

[24] Tan H, Jain A, Voznyy O, Lan X, De Arquer FPG, Fan JZ. Science. Science. 2017;355:722. 28154242 10.1126/science.aai9081

[25] Tao C, Neutzner S, Colella L, Marras S, Srimath Kandada AR, Gandini M. Energy and Environmental Science. Energy Environ Sci. 2015;8:2365.

[26] Zhang F, Shi W, Luo J, Pellet N, Yi C, Li X. Adv Mater. 2017;29:1606806. 10.1002/adma.201606806 28240401

[27] Wang F, Shimazaki A, Yang F, Kanahashi K, Matsuki K, Miyauchi Y. J Phys Chem C. 2017;121:1562.

[28] Koushik D, Verhees W, Kuang Y, Veenstra S, Zhang D, Verheijen M. Energy Environ Sci. 2017;10:91.

[29] Mahmud MA, Duong T, Yin Y, Pham HT, Walter D, Peng J. Advanced Functional Materials. 2020;30:1907962.

[30] Mahmud M, Duong T, Yin Y, Peng J, Wu Y, Lu T. Small. 2020;16:2005022. 10.1002/smll.202005022 33201580

[31] Zhao P, Kim BJ, Jung HS. Mater Today Energy. Mater Today Energy. 2018;7:267.

[32] Zhang F, Xiao C, Chen X, Larson BW, Harvey SP, Berry JJ. Joule. 2019;3:1452.

[33] Zhang F, Shi W, Luo J, Pellet N, Yi C, Li X. Adv Mater. 2017;29:1606806. 10.1002/adma.201606806 28240401

[34] Eperon GE, Leijtens T, Bush KA, Prasanna R, Green T, Wang JTW. Science. Science. 2016;354:861. 27856902 10.1126/science.aaf9717

[35] Grancini G, Roldán-Carmona C, Zimmermann I, Mosconi E, Lee X, Martineau D. Nature Communications. 2017;8:1. 10.1038/ncomms15684 PMC5461484 28569749

[36] Zheng X, Troughton J, Gasparini N, Lin Y, Wei M, Hou Y. Joule. 2019;3:1963.

[37] Gao L, Spanopoulos I, Ke W, Huang S, Hadar I, Chen L. ACS Energy Lett. 2019;4:1763.

[38] Fan J, Ma Y, Zhang C, Liu C, Li W, Schropp REI. Advanced Energy Materials. 2018;8:1703421.

[39] Hu Y, Qiu T, Bai F, Ruan W, Zhang S. Adv Energy Mater. Adv Energy Mater. 2018;8:1703620.

[40] Zhou Y, Xue H, Jia YH, Brocks G, Tao S, Zhao N. Advanced Functional Materials. 2019;29:1.

[41] Kim H, Lee SU, Lee DY, Paik MJ, Na H, Lee J. Adv Energy Mater. Adv Energy Mater. 2019;9:1902740.

[42] Bu T, Li J, Huang W, Mao W, Zheng F, Bi P. Journal of Materials Chemistry A. 2019;7:6793.

[43] Kim M, Kim GH, Lee TK, Choi IW, Choi HW, Jo Y. Joule. 2019;3:2179.

[44] Paritmongkol W, Dahod NS, Stollmann A, Mao N, Settens C, Zheng SL. Chem Mater. 2019;31:5592. 10.1021/acs.jpclett.9b00983 31066277

[45] Park IH, Zhang Q, Kwon KC, Zhu Z, Yu W, Leng K. J Am Chem Soc. 2019;141:15972. 31522501 10.1021/jacs.9b07776

[46] Zhang Y, Wang P, Tang MC, Barrit D, Ke W, Liu J. J Am Chem Soc. 2019;141:2684. 30648861 10.1021/jacs.8b13104

[47] Soe CMM, Stoumpos CC, Kepenekian M, Traoré B, Tsai H, Nie W. J Am Chem Soc. 2017;139:16297. 29095597 10.1021/jacs.7b09096

[48] Zhang F, Kim DH, Zhu K. Curr Opin Electrochem. 2018;11:105.

[49] Yoo JJ, Wieghold S, Sponseller MC, Chua MR, Bertram SN, Hartono NTP. Energy Environ Sci. 2019;12:2192.

[50] Ma C, Leng C, Ji Y, Wei X, Sun K, Tang L, et al. Nanoscale. 2016;8:18309. 27714126 10.1039/c6nr04741f

[51] Hu Y, Schlipf J, Wussler M, Petrus ML, Jaegermann W, Bein T. ACS Nano. 2016;10:5999. 27228558 10.1021/acsnano.6b01535

[52] Koh TM, Shanmugam V, Guo X, Lim SS, Filonik O, Herzig EM. Journal of Materials Chemistry A. 2018;6:2122.

[53] Duong T, Pham H, Yin Y, Peng J, Mahmud MA, Wu YL. Journal of Materials Chemistry A. 2021;9:18454.

[54] Gharibzadeh S, Abdollahi Nejand B, Jakoby M, Abzieher T, Hauschild D, Moghadamzadeh S. Adv Energy Mater. Adv Energy Mater. 2019;9:1803699.

[55] Duong T, Pham H, Kho TC, Phang P, Fong KC, Yan D, et al. Adv Energy Mater. 2020;10:1903553.

[56] Stolterfoht M, Wolff CM, Márquez JA, Zhang S, Hages CJ, Rothhardt D. Nature Energy. 2018;3:847.

[57] Schulz P. ACS Energy Lett. 2018;3:1287.

[58] Mahmud MA, Pham HT, Duong T, Yin Y, Peng J, Wu Y. Advanced Functional Materials. 2021;31:2104251. doi: 10.1002/adfm.202104251

[59] Chavan RD, Prochowicz D, Tavakoli MM, Yadav P, Hong CK. Adv Mater Interfaces. 2020;7:1.

[60] Pham ND, Tiong VT, Yao D, Martens W, Guerrero A, Bisquert J. Nano Energy. 2017;41:476.

[61] Teale S, Degani M, Chen B, Sargent EH, Grancini G. Molecular cation and low-dimensional perovskite surface passivation in perovskite solar cells. Nat Energy. 2024;9(7):779–92. doi: 10.1038/s41560-024-01529-3

[62] Jeong J, Chawanpunyawat T, Kim M, Sláma V, Lempesis N, Agosta L, et al. Carbazole Treated Waterproof Perovskite Films with Improved Solar Cell Performance. Advanced Energy Materials. 2024;15(2). doi: 10.1002/aenm.202401965

[63] Almalki M, Alotaibi MH, Alanazi AQ, Eickemeyer FT, Alenzi SM, Alzahrani YA. Interfacial Modulation through Mixed-Dimensional Heterostructures for Efficient and Hole Conductor-Free Perovskite Solar Cells. Advanced Functional Materials. 2024;34:2309789.

[64] Gan Y, Sun J, Guo P, Jiang H, Li J, Zhu H, et al. Advances in the research of carbon electrodes for perovskite solar cells. Dalton Trans. 2023;52(45):16558–77. doi: 10.1039/d3dt03136e 37831439

[65] Khadka DB, Shirai Y, Yanagida M, Ota H, Lyalin A, Taketsugu T, et al. Defect passivation in methylammonium/bromine free inverted perovskite solar cells using charge-modulated molecular bonding. Nat Commun. 2024;15(1):882. doi: 10.1038/s41467-024-45228-9 38287031 PMC10824754

[66] Chen S, Shen N, Zhang L, Kong W, Zhang L, Cheng C. Journal of Materials Chemistry A. 2019;7:9542.

[67] Tan S, Zhou N, Chen Y, Li L, Liu G, Liu P. Advanced Energy Materials. 2019;9:1803024.

[68] Qiu J, Xia Y, Zheng Y, Hui W, Gu H, Yuan W. ACS Energy Lett. 2019;4:1513.

[69] Zhang Y, Chen J, Lian X, Qin M, Li J, Andersen TR. Small Methods. Small Methods. 2019;3:1.

[70] Nazarenko O, Kotyrba MR, Yakunin S, Aebli M, Rainò G, Benin BM. J Am Chem Soc. 2018;140:3850. 29502407 10.1021/jacs.8b00194 PMC5867663

[71] Jiang Q, Zhao Y, Zhang X, Yang X, Chen Y, Chu Z, et al. Surface passivation of perovskite film for efficient solar cells. Nat Photonics. 2019;13(7):460–6. doi: 10.1038/s41566-019-0398-2

[72] Mozaffari N, Duong T, Shehata MM, Bui AD, Pham HT, Yin Y. Above 23% efficiency by binary surface passivation of perovskite solar cells using guanidinium and octylammonium spacer cations. Sol RRL. 2022;6:2200355.

[73] Osman MM, El-naggar AM, Alanazi AQ, Aldhafiri AM, Albassam AA. Development of Perovskite (MACl)_0.33_FA_0.99_MA_0.01_Pb(I_0.99_Br_0.01_)_3_ Solar Cells via n-Octylammonium Iodide Surface Passivation. Nanomaterials. 2023;13:1492. doi: 10.3390/nano13091492 37177037 PMC10179917

[74] Jiang Q, Zhao Y, Zhang X, Yang X, Chen Y, Chu Z, et al. Nat Photonics. 2019;13:460.

