# Correction: Enhancing perovskite solar cells efficiency via dual surface passivation

**DOI:** 10.1371/journal.pone.0356803

**Published:** 2026-08-20

**Authors:** Refka Sai, Shrouq H. Aleithan

The Funding statement for this article is incorrect. The correct Funding statement is as follows: This work was supported through the Annual Funding track by the Deanship of Scientific Research, Vice Presidency for Graduate Studies and Scientific Research, King Faisal University, Saudi Arabia [KFU262906].
